# Genome-Wide Identification of Ralstonia solanacearum Genes Required for Survival in Tomato Plants

**DOI:** 10.1128/mSystems.00838-21

**Published:** 2021-10-12

**Authors:** Yaxing Su, Yanan Xu, Hailing Liang, Gaoqing Yuan, Xiaogang Wu, Dehong Zheng

**Affiliations:** a State Key Laboratory for Conservation and Utilization of Subtropical Agro-bioresources, Guangxi Key Laboratory of Agro-Environment and Agro-Product Safety, College of Agriculture, Guangxi University, Nanning, People’s Republic of China; b Pharmaceutical College, Guangxi Medical University, Nanning, People’s Republic of China; University of Dundee

**Keywords:** pathogenic bacteria, pathogenicity, *Ralstonia solanacearum*, transposon sequencing

## Abstract

Ralstonia solanacearum is an extremely destructive phytopathogenic bacterium for which there is no effective control method. Though many pathogenic factors have been identified, the survival strategies of R. solanacearum in host plants remain unclear. Transposon insertion sequencing (Tn-seq) is a high-throughput genetic screening technology. This study conducted a Tn-seq analysis using the *in planta* environment as selective pressure to identify R. solanacearum genes required for survival in tomato plants. One hundred thirty genes were identified as putative genes required for survival in tomato plants. Sixty-three of these genes were classified into four Clusters of Orthologous Groups categories. The absence of genes that encode the outer membrane lipoprotein LolB (RS_RS01965) or the membrane protein RS_RS04475 severely decreased the *in planta* fitness of R. solanacearum. *RS_RS09970* and *RS_RS04490* are involved in tryptophan and serine biosynthesis, respectively. Mutants that lack *RS_RS09970* or *RS_RS04490* did not cause any wilt symptoms in susceptible tomato plants. These results confirmed the importance of genes related to “cell wall/membrane/envelope biogenesis” and “amino acid transport and metabolism” for survival in plants. The gene encoding NADH-quinone oxidoreductase subunit B (*RS_RS10340*) is one of the 13 identified genes involved in “energy production and conversion,” and the Clp protease gene (*RS_RS08645*) is one of the 11 identified genes assigned to “posttranslational modification, protein turnover, and chaperones.” Both genes were confirmed to be required for survival in plants. In conclusion, this study globally identified and validated R. solanacearum genes required for survival in tomato plants and provided essential information for a more complete view of the pathogenic mechanism of R. solanacearum.

**IMPORTANCE** Tomato plant xylem is a nutritionally limiting and dynamically changing habitat. Studies on how R. solanacearum survives in this hostile environment are important for our full understanding of the pathogenic mechanism of this bacterium. Though many omics approaches have been employed to study *in planta* survival strategies, the direct genome-wide identification of R. solanacearum genes required for survival in plants is still lacking. This study performed a Tn-seq analysis in R. solanacearum and revealed that genes in the categories “cell wall/membrane/envelope biogenesis,” “amino acid transport and metabolism,” “energy production and conversion,” “posttranslational modification, protein turnover, chaperones” and others play important roles in the survival of R. solanacearum in tomato plants.

## INTRODUCTION

Ralstonia solanacearum is an aerobic, motile Gram-negative bacterium with a polar flagellar tuft. This soilborne bacterium is probably the most destructive plant-pathogenic bacterium, infecting more than 200 plant species in over 50 families over a broad geographical range (1). The host plants of R. solanacearum include tomato, tobacco, potato, peanut, and many other important commercial crops (2, 3). R. solanacearum is extremely damaging and has caused an estimated US$1 billion loss each year worldwide on potatoes alone because of the lack of an effective control method (4). A better understanding of the pathogenic mechanism is an essential precondition for controlling plant diseases caused by R. solanacearum.

As in most Gram-negative animal- and plant-pathogenic bacteria, the type III secretion system and effectors secreted by this system are the major pathogenicity determinants in R. solanacearum (5). The absence of the structural proteins of type III secretion system apparatus (6) or the key regulators of type III secretion system genes, such as HrpB (7), abolishes the pathogenicity of R. solanacearum. Moreover, type III secretion system effectors are the key host range factors. The *gala7* gene extends the host range of R. solanacearum GMI1000 to include the legume Medicago truncatula (8). High-molecular-mass exopolysaccharide (EPS) is another important virulence determinant for R. solanacearum. This EPS contributes to rapid systemic colonization by R. solanacearum and wilt symptoms in susceptible hosts; mutants that lack *eps* genes cannot cause disease symptoms on host plants (9). Moreover, cell wall-degrading enzymes secreted by the type II secretion system, motility, resistance to stresses within the host plant, nutrient-scavenging systems, and varied regulatory networks play important roles in infection by R. solanacearum (10).

Besides targeted genetic studies, omics approaches have been used to identify pathogenicity genes in R. solanacearum. The most used strategy is to identify genes induced during plant infection. Brown and Allen revealed 153 *in planta*-expressed genes using *in vivo* expression technology and suggested that R. solanacearum confronts and overcomes stressful and nutrient-poor environments (11). An *in planta* transcriptome study revealed that about 12% of R. solanacearum transcriptomes were remarkably altered *in planta* compared with in rich medium and that the absence of the sucrose uptake and catabolism gene *scrA* impaired the virulence of R. solanacearum on tomato, potato, and Solanum dulcamara (12). *In silico* or experimental evolution is another strategy used to screen pathogenicity genes in R. solanacearum. Forty-nine genes in 37 R. solanacearum genomes were identified as nonneutrally evolving and maybe virulence related using Tajima’s D population genetic test (13). The multihost experimental evolution of selected independent mutations in the regulatory gene *efpR* revealed that it is a determinant conditioning host adaptation of R. solanacearum (14).

Transposon insertion sequencing (Tn-seq) is a high-throughput approach that couples genome-wide transposon mutagenesis with next-generation sequencing (15, 16). Tn-seq can be used to identify genes that contribute to bacterial survival under the selective pressure of interest (17). Tn-seq has been applied to identify genes important for *in vivo* survival in several plant-pathogenic bacteria using the host environment as the selective pressure. A Tn-seq study inoculated the transposon mutant library of Dickeya dadantii in chicory plant and recovered the mutants from rotten tissue after 2 days; this Tn-seq study revealed that about 100 genes contribute to the survival of *D. dadantii* in chicory plants (18). A total of 486 genes in Pantoea stewartii subsp. *stewartia* are essential for survival in corn xylem, as determined through a Tn-seq analysis (19).

A Tn-seq analysis of R. solanacearum in tomato plants was conducted in the present study to acquire a more complete view of the pathogenic mechanism of R. solanacearum. The transposon insertion library was injected into the tomato plant stem and recovered 5 days postinoculation. The transposon interruption of 130 genes reduced the relative fitness of R. solanacearum within tomato plants, providing putative genes required for R. solanacearum survival in tomato plants. Furthermore, targeted gene deletion, pathogenicity assay, *in vivo* colonization assay, and competition index determination were performed to validate the results of Tn-seq.

## RESULTS

### Tn-seq analysis to identify putative genes required for survival in tomato plants.

**FIG 1:**
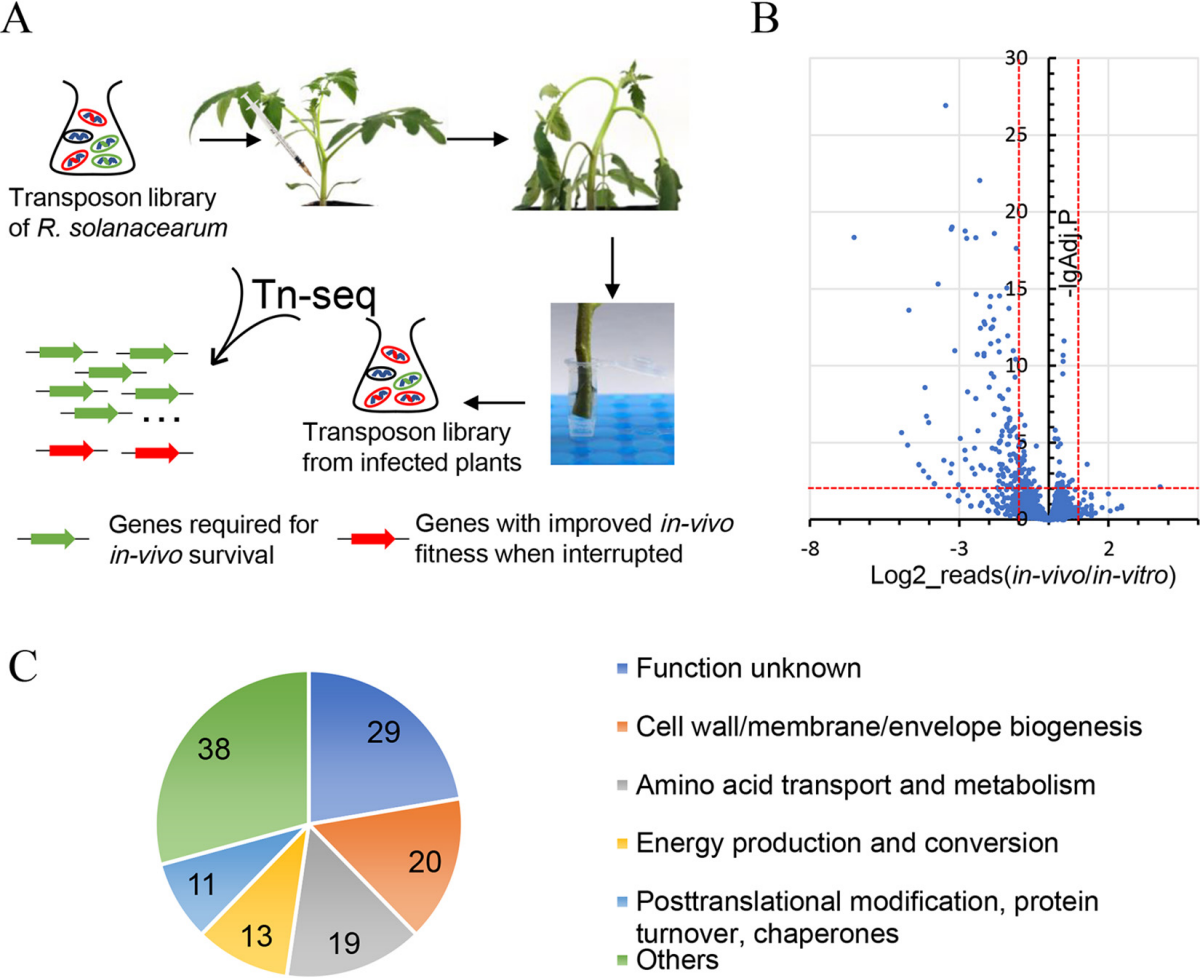
Tn-seq analysis of R. solanacearum genes required for survival in tomato plants. (A) Schematic diagram of Tn-seq analysis in this study. (B) Gene essentiality for *in vivo* survival. Each dot represents a gene, plotted by the ratio (*in vivo*/*in vitro*) of reads mapped within this gene on the horizontal axis and the -lg (adjusted *P* value) on the vertical. A ratio_reads (*in vivo*/*in vitro*) value of <0.5 or >2 with an adjusted *P* (proportions_reads) value of <0.01 was set as the threshold value to identify genes required for survival in tomato plants or genes resulting in improved *in vivo* fitness when interrupted. (C) Numbers of genes required for survival in tomato plants classified by COG categories.

We previously constructed a near-saturated transposon insertion library of R. solanacearum GMI1000 with approximately 240,000 individual insertion mutants, covering 70.44% to 80.96% of all potential insertion sites (20). An *in planta* Tn-seq analysis was conducted using this near-saturated transposon insertion library to identify R. solanacearum genes essential for *in planta* survival. As shown in Fig. 1A, the transposon insertion library of R. solanacearum was activated and injected into the tomato plant stem (21). The library was then recovered from the stems of 32 inoculated tomato plants 5 days postinoculation, when the wilting symptom scores of most tomato plants were 3 or 2. The transposon insertion libraries recovered from all tomato plants were then pooled. The total genome DNAs of transposon insertion libraries before (*in vitro*) and after (*in vivo*) infection were extracted and split into two groups for technical replicates. The DNA samples were then subjected to Tn-seq to identify the relative abundance of each insertion mutant under the stress of the *in planta* environment.

The correlation between technical replicates is analyzed and visualized in Fig. S1 in the supplemental material. The correlation coefficients for the *in vivo* and *in vitro* replicates were 1.00 and 0.99, respectively, which indicate the reliability and repeatability of this analysis. As shown in Table 1, 6,858,640 reads of *in-vivo*1 were mapped to the chromosome (NC_003295.1) of R. solanacearum strain GMI1000, and 4,636,990 reads of *in-vivo*1 were mapped to the megaplasmid (NC_003296.1). These reads hit 135,231 unique locations with 101,663 locations within genes. The output parameters of *in-vivo*2 were similar to that of *in-vivo*1. However, more reads were mapped to the chromosome, and fewer reads were mapped to the megaplasmid for the *in vitro* treatments than for the *in vivo* treatments. The transposon interruption of a gene essential for survival in plants would reduce the relative fitness of a mutant within tomato plants and result in fewer reads mapped to this gene. As shown in Fig. 1B, 130 genes in R. solanacearum strain GMI1000 were identified as putative genes required for survival in tomato plants when the threshold value was set to a ratio_reads (*in vivo*/*in vitro*) value of <0.5 with an adjusted *P* (proportions_reads) value of <0.01. The interruption of two genes (*RS_RS05450* and *RS_RS05405*) increased the ratio_reads (*in vivo*/*in vitro*) value by more than 2-fold (Table S1). Interestingly, 117 of these 132 genes that contribute to the survival in tomato plants are located in the chromosome of R. solanacearum strain GMI1000.

**TABLE 1:** Output parameters of the *in planta* Tn-seq analysis

Treatment	No. of:		
	Mapped reads (chromosome + megaplasmid)	Unique hits	Unique hits within genes
*in-vivo*1	6,858,640 + 4,636,990	135,231	101,663
*in-vivo*2	6,835,235 + 4,622,666	133,781	100,365
*in-vitro*1	7,014,722 + 4,093,910	174,518	131,536
*in-vitro*2	7,196,108 + 4,206,360	175,751	132,999

10.1128/mSystems.00838-21.1 TABLE S1 Gene essentiality for *in vivo* survival. Download Table S1, XLSX file, 0.03 MB. Copyright © 2021 Su et al. 2021 Su et al. https://creativecommons.org/licenses/by/4.0/ This content is distributed under the terms of the Creative Commons Attribution 4.0 International license.

10.1128/mSystems.00838-21.3 FIG S1 Coefficient of read coverages for Tn-seq technological replicates (scatter plot) and all Tn-seq samples (heat map). Download FIG S1, TIF file, 0.1 MB. Copyright © 2021 Su et al. 2021 Su et al. https://creativecommons.org/licenses/by/4.0/ This content is distributed under the terms of the Creative Commons Attribution 4.0 International license.

We classified these genes into Clusters of Orthologous Groups of proteins (COG) categories to obtain an overview of the genes required for survival in tomato plants. As shown in Fig. 1C, 29 of these 130 genes were annotated as “function unknown” or were not mapped to COG categories. Twenty genes involved in “cell wall/membrane/envelope biogenesis” are required for survival in tomato plants. The genes assigned to “amino acid transport and metabolism,” “energy production and conversion,” and “posttranslational modification, protein turnover, chaperones” play important roles in the survival of R. solanacearum in tomato plants.

### Cell wall/membrane/envelope biogenesis-related genes required for survival in tomato plants.

**FIG 2:**
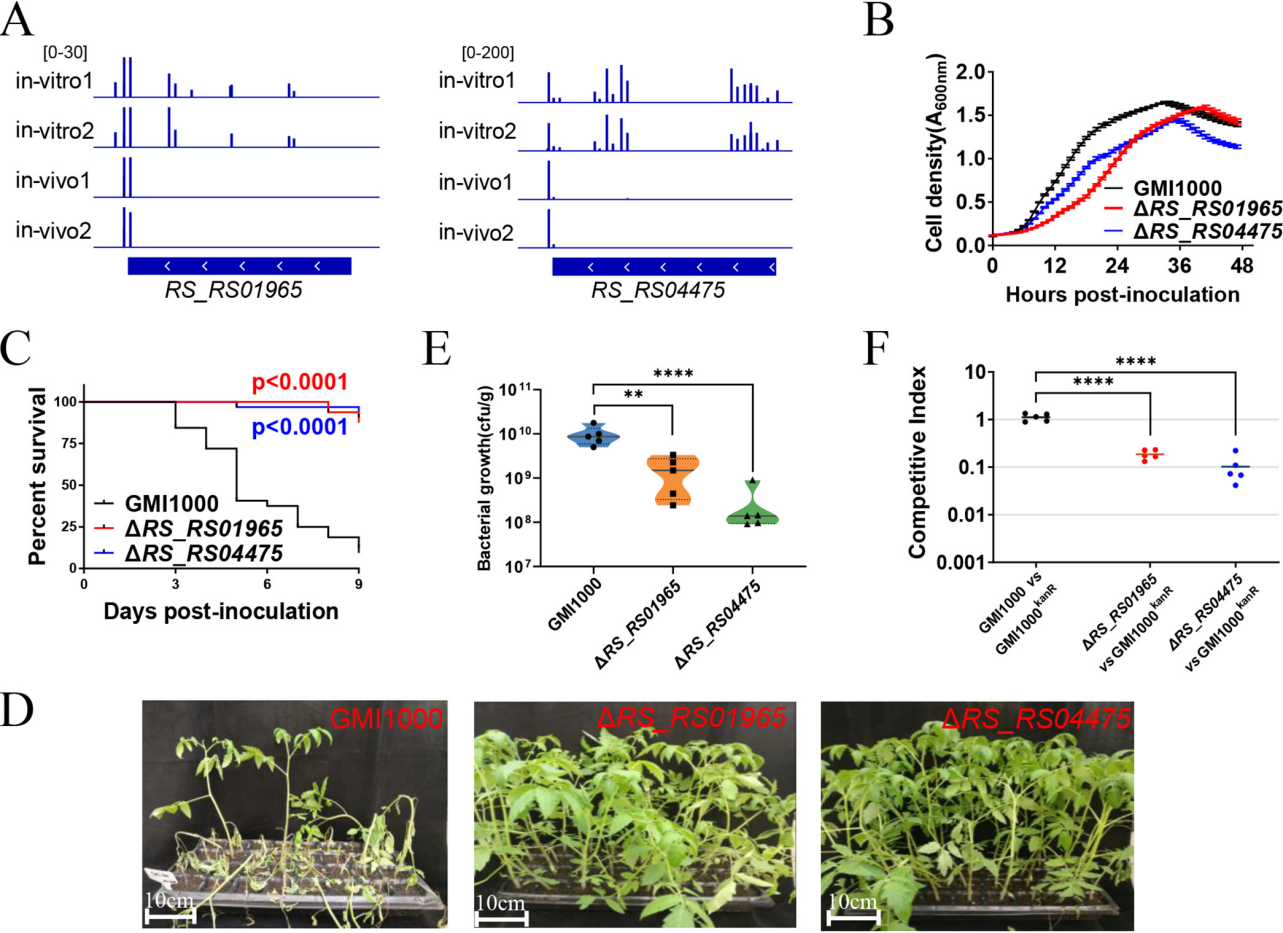
*RS_RS01965* and *RS_RS04475* are required for survival in tomato plants. (A) Transposon insertion distribution within *RS_RS01965* and *RS_RS04475* of transposon insertion libraries *in vivo* and *in vitro*. (B) Growth of the Δ*RS_RS01965* mutant, the Δ*RS_RS04475* mutant, and wild-type strain GMI1000 in BG medium. (C) Survival curve of tomato plants inoculated with the Δ*RS_RS01965* mutant, the Δ*RS_RS04475* mutant, and wild-type strain GMI1000. Kaplan-Meier survival analysis with the Gehan-Breslow-Wilcoxon method was used to compare the pathogenicity between the mutant and wild-type strains. A *P* value of <0.05 was considered significant. (D) Bacterial wilt symptoms of tomato plants 9 days after inoculation with the Δ*RS_RS01965* mutant, the Δ*RS_RS04475* mutant, and wild-type strain GMI1000. (E) Colonization of Δ*RS_RS01965* and Δ*RS_RS04475* mutants. The CFU of R. solanacearum strains in 1 g tomato plant stem were counted 5 days postinoculation. Asterisks indicate significant differences (**, *P* < 0.01; ****, *P* < 0.0001; *t* test). F. *In vivo* competitive index of Δ*RS_RS01965* and Δ*RS_RS04475* mutants. R. solanacearum mutants and GMI1000^Kanr^ were coinoculated into the stems of tomato plants. The competitive index was measured 5 days postinoculation. Asterisks indicate significant differences (****, *P* < 0.0001; *t* test).

Twenty genes assigned to “cell wall/membrane/envelope biogenesis” were found to be vital for the survival of R. solanacearum in tomato plants by Tn-seq. *RS_RS01965* encodes the outer membrane lipoprotein LolB, which is a component of the LolABCDE system, responsible for sorting and localizing lipoprotein. The absence of LolA in the plant pathogen Xanthomonas campestris pv. *campestris* reduced the pathogen’s attachment, extracellular enzyme production, and virulence (22). As shown in Fig. 2A and Table S1, 6.5 unique hits were found within *RS_RS01965* mapped by 26 weighted reads in average before infection, but no transposon insertion mutant of *RS_RS01965* was detected from the transposon insertion library recovered from the infected tomato. *RS_RS04475* encodes a membrane protein. The relative abundance of the *RS_RS04475* mutant was 0.039 times higher in tomato plants than in *in vitro* (Fig. 2A). *RS_RS01965* and *RS_RS04475* were deleted in frame individually to validate the contribution of these genes in this category.

The *RS_RS01965* (Δ*RS_RS01965*) and *RS_RS04475* (Δ*RS_RS04475*) deletion mutants exhibited impaired growth in rich BG medium (Fig. 2B). These two mutants were then injected into the tomato stem to evaluate the pathogenic contribution of *RS_RS01965* and *RS_RS04475*. As shown in Fig. 2C and D, almost all the tested tomato plants were wilted 9 days postinoculation of R. solanacearum wild-type strain GMI1000, whereas almost all tomato plants inoculated with the Δ*RS_RS01965* or Δ*RS_RS04475* mutant survived. R. solanacearum cells (10^5^ CFU) were injected into the tomato stem to assay the colonization of these mutants. As shown in Fig. 2E, 10^9.9^ CFU R. solanacearum were detected in 1 g tomato plant stem 5 days after inoculation of the wild-type strain. However, this number was 10^9.0^ and 10^8.2^ for the Δ*RS_RS01965* and Δ*RS_RS04475* mutants, respectively. Moreover, the competitive index was measured to confirm the results of Tn-seq. The Δ*RS_RS01965* and Δ*RS_RS04475* strains were outcompeted by GMI1000^Kanr^ with competitive index values of 0.19 and 0.10, respectively (Fig. 2F). These results indicated that *RS_RS01965* and *RS_RS04475* are essential for the survival of R. solanacearum in tomato plants.

### Amino acid transport and metabolism-related genes required for survival in tomato plants.

Nineteen amino acid transport and metabolism genes were identified as essential for survival in tomato plants by Tn-seq (Table S1). Ten of these genes were mapped to the pathway of “phenylalanine, tyrosine and tryptophan biosynthesis” in the Kyoto Encyclopedia of Genes and Genomes (KEGG) database (Fig. S2). Four (*RS_RS14885*, *RS_RS07885*, *RS_RS13320*, and *RS_RS04510*) of these 10 genes are involved in the shikimate pathway, which synthesizes chorismite, an important biochemical intermediate for amino acid biosynthesis. Four genes are responsible for tryptophan biosynthesis, including tryptophan synthase subunit alpha (*RS_RS09955*; TrpA), tryptophan synthase subunit beta (*RS_RS09965*; TrpB), anthranilate synthase component I (*RS_RS14430*; TrpE), and phosphoribosylanthranilate isomerase (*RS_RS09970*; TrpF). Two genes (*RS_RS14785* and *RS_RS05000*) are involved in the biosynthesis of phenylalanine and tyrosine (Fig. S2). In addition, genes involved in the biosynthesis of serine (*RS_RS08265* and *RS_RS04490*), cysteine (*RS_RS05790*), methionine (*RS_RS00135*), and lysine (*RS_RS05700*) were also identified as essential for the survival of R. solanacearum in tomato plants.

10.1128/mSystems.00838-21.4 FIG S2 Genes involved in the “phenylalanine, tyrosine, and tryptophan biosynthesis” pathway are required for survival in tomato plants. Download FIG S2, TIF file, 0.7 MB. Copyright © 2021 Su et al. 2021 Su et al. https://creativecommons.org/licenses/by/4.0/ This content is distributed under the terms of the Creative Commons Attribution 4.0 International license.

**FIG 3:**
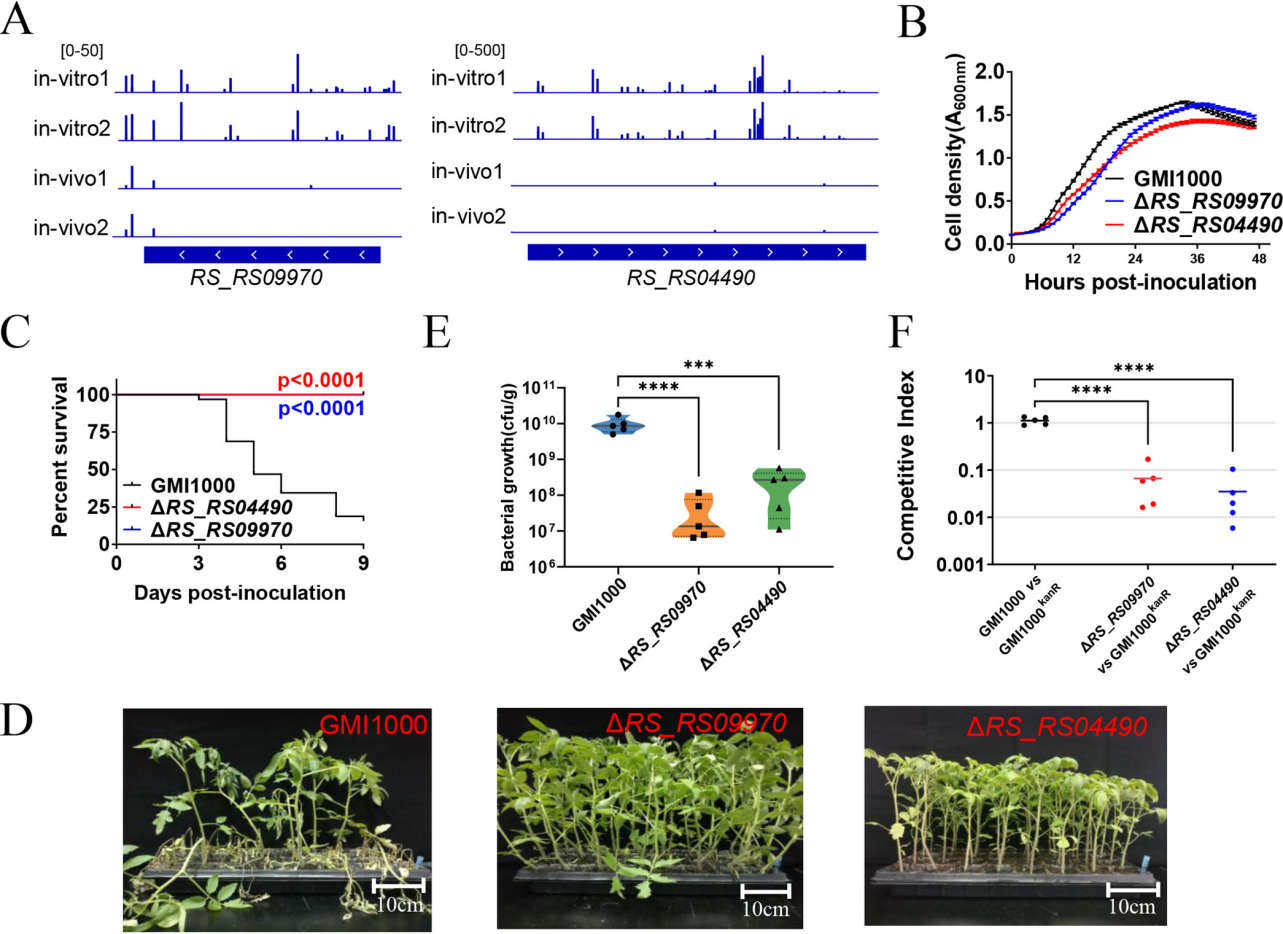
*RS_RS09970* and *RS_RS04490* are required for survival in tomato plants. (A) Transposon insertion distribution within *RS_RS09970* and *RS_RS04490* of transposon insertion libraries *in vivo* and *in vitro*. (B) Growth of the Δ*RS_RS09970* mutant, the Δ*RS_RS04490* mutant, and wild-type strain GMI1000 in BG medium. (C) Survival curve of tomato plants inoculated with the Δ*RS_RS09970* mutant, the Δ*RS_RS04490* mutant, and wild-type strain GMI1000. Kaplan-Meier survival analysis with the Gehan-Breslow-Wilcoxon method was used to compare pathogenicity between the mutant and wild-type strains. A *P* value of <0.05 was considered significant. (D) Bacterial wilt symptoms of tomato plants 9 days after inoculation of the Δ*RS_RS09970* mutant, the Δ*RS_RS04490* mutant, and wild-type strain GMI1000. (E) Colonization of Δ*RS_RS09970* and Δ*RS_RS04490* mutants. The CFU of R. solanacearum strains in 1 g tomato plant stem were counted 5 days postinoculation. Asterisks indicate significant differences (***, *P* < 0.001; ****, *P* < 0.0001; *t* test). (F) *In vivo* competitive index of Δ*RS_RS09970* and Δ*RS_RS04490* mutants. R. solanacearum mutants and GMI1000^Kanr^ were coinoculated into the stems of tomato plants. The competitive index was measured 5 days postinoculation. Asterisks indicate significant differences (****, *P* < 0.0001; *t* test).

Two genes related to tryptophan and serine biosynthesis were targeted to verify their pathogenic contribution in R. solanacearum. As shown in Fig. 3A and Table S1, 13 unique insertion locations in *RS_RS09970* were mapped by 52 weighted reads in average before infection, whereas the transposon insertion mutant of *RS_RS09970* was hardly detected from the library after tomato plant infection. The relative abundance of the *RS_RS04490* mutant was also sharply reduced after infection (Fig. 3A and Table S1). *RS_RS09970* and *RS_RS04490* were then deleted individually. As shown in Fig. 3B, the deletion of *RS_RS09970* or *RS_RS04490* slightly impaired the growth of R. solanacearum in rich BG medium. The functions of *RS_RS09970* and *RS_RS04490* in amino acid biosynthesis were verified. Δ*RS_RS09970* and Δ*RS_RS04490* mutants cannot grow in minimal Fahraeus medium. The auxotrophs of the Δ*RS_RS09970* and Δ*RS_RS04490* mutants could be rescued by supplementation with tryptophan and serine, respectively (Fig. S3). The pathogenicity of these two mutants was then assayed by stem injection. As shown in Fig. 3C and D, Δ*RS_RS09970* and Δ*RS_RS04490* mutants did not cause any disease symptoms on tomato plants. Moreover, colonization by the Δ*RS_RS09970* and Δ*RS_RS04490* mutants was remarkably weaker than that of the wild-type strain GMI1000 (Fig. 3E). The Δ*RS_RS09970* and Δ*RS_RS04490* mutants were outcompeted when coinoculated with GMI1000^Kanr^ into tomato plants with competitive index values of 0.07 and 0.04, respectively (Fig. 3F). These results indicate that *RS_RS09970* and *RS_RS04490* are essential for fitness in tomato plants.

10.1128/mSystems.00838-21.5 FIG S3 The Δ*RS_RS09970* and Δ*RS_RS04490* mutants are auxotrophs. (A) Growth of the Δ*RS_RS09970* mutant, the Δ*RS_RS04490* mutant, and wild-type GMI1000 in liquid minimal Fahraeus medium with or without tryptophan (Trp) or serine (Ser). The growth (*A*_600_) of each R. solanacearum strain in a given medium was monitored every hour via Bioscreen C Pro. The growth was indicated by the means of three biological replicates. The error bars indicates standard deviations. (B) Growth of the Δ*RS_RS09970* mutant, the Δ*RS_RS04490* mutant, and wild-type strain GMI1000 on Fahraeus agar medium with or without tryptophan (Trp) or serine (Ser). Gradient-diluted R. solanacearum strains were cultured on Fahraeus medium and photographed 24 h postinoculation. Download FIG S3, TIF file, 0.8 MB. Copyright © 2021 Su et al. 2021 Su et al. https://creativecommons.org/licenses/by/4.0/ This content is distributed under the terms of the Creative Commons Attribution 4.0 International license.

### Energy production and conversion-related genes required for survival in tomato plants.

**FIG 4:**
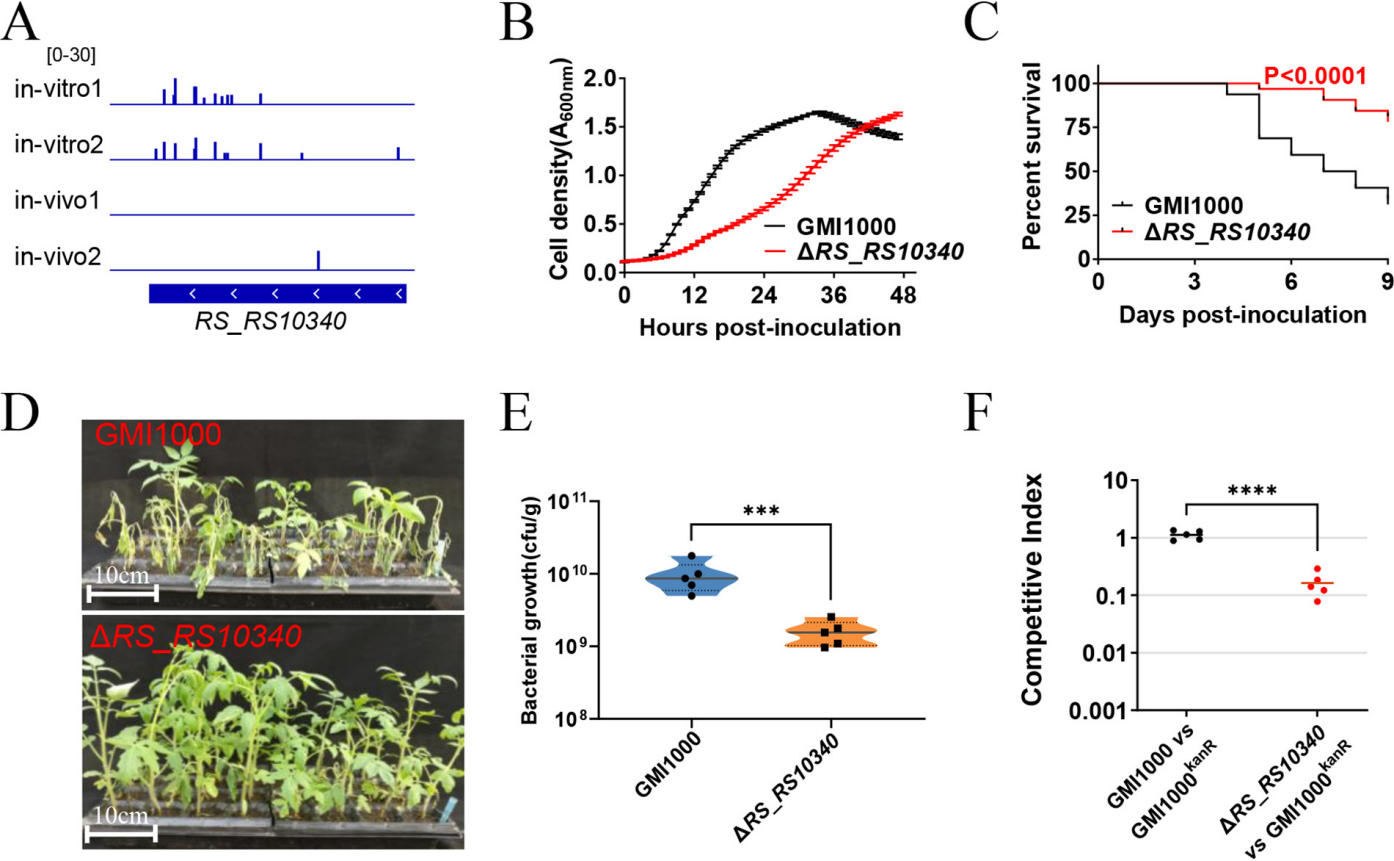
*RS_RS10340* is required for survival in tomato plants. (A) Transposon insertion distribution within *RS_RS10340* of transposon insertion libraries *in vivo* and *in vitro*. (B) Growth of the Δ*RS_RS10340* mutant and wild-type strain GMI1000 in BG medium. (C) Survival curve of tomato plants inoculated with the Δ*RS_RS10340* mutant and wild-type strain GMI1000. Kaplan-Meier survival analysis with the Gehan-Breslow-Wilcoxon method was used to compare pathogenicity between the mutant and wild-type strains. A *P* value of <0.05 was considered significant. (D) The bacterial wilt symptom of tomato plants 9 days postinoculation of the Δ*RS_RS10340* mutant and wild-type strain GMI1000. (E) Colonization of the Δ*RS_RS10340* mutant. The CFU of R. solanacearum strains in 1 g tomato plant stem were counted 5 days postinoculation. Asterisks indicate significant differences (***, *P* < 0.001; *t* test). (F) *In vivo* competitive index of the Δ*RS_RS10340* mutant. The Δ*RS_RS10340* mutant and GMI1000^Kanr^ were coinoculated into the stems of tomato plants. The competitive index was measured 5 days postinoculation. Asterisks indicate significant differences (****, *P* < 0.0001; *t* test).

The transposon insertion mutants of 13 energy production and conversion-related genes were less frequently detected in the transposon insertion libraries recovered from the inoculated tomato plants. Thus, energy production- and conversion-related genes are more required for R. solanacearum to survive in tomato plants than in rich medium. Seven of these 13 genes are NADH-quinone oxidoreductase subunit-encoding genes (Table S1). For example, the relative abundance of a NADH-quinone oxidoreductase subunit B (*RS_RS10340*) mutant in *in vivo* libraries was 0.06 times higher than that in *in vitro* libraries (Fig. 4A and Table S1). *RS_RS10340* was then deleted in frame to verify the importance of energy production- and conversion-related genes for survival in tomato plants.

As shown in Fig. 4B, the final concentration of *RS_RS10340* deletion mutant in rich BG medium was similar to that of the wild-type strain GMI1000, though the deletion of *RS_RS10340* seriously slowed the growth of R. solanacearum. The Δ*RS_RS10340* mutant and wild-type strain GMI1000 were inoculated into tomato plants by injection to assay pathogenicity. As shown in Fig. 4C and D, only 34% of tomato plants survived 9 days after inoculation with wild-type strain, whereas 81% of plants survived 9 days after inoculation with the Δ*RS_RS10340* mutant. Moreover, the absence of *RS_RS10340* remarkably attenuated the growth of R. solanacearum in tomato plants (Fig. 4E). The competition assay confirmed that the wild-type strain overgrew the Δ*RS_RS10340* mutant in tomato plants (Fig. 4F). Thus, *RS_RS10340* is required for the survival of R. solanacearum in tomato plants.

### Posttranslational modification, protein turnover, and chaperone-related genes required for survival in tomato plants.

Eleven of the genes required for R. solanacearum survival in tomato plants were categorized as “posttranslational modification, protein turnover, chaperones” (Table S1). Four of these 11 genes, namely, those encoding the FtsH protease activity modulator HflK (*RS_RS06120*), protease modulator HflC (*RS_RS06125*), ATP-dependent Clp protease proteolytic subunit (*RS_RS08645*; *clpP*), and ATP-dependent Clp protease ATP-binding subunit ClpX (*RS_RS08650*), are involved in proteolysis. ClpP protease plays an important role in the proteostasis of prokaryotic cells and eukaryotic organelles (23). ClpP was found to be essential for the full virulence of Salmonella enterica serovar Typhimurium, Staphylococcus aureus, and the phytopathogen X. campestris (24–26). Thus, *clpP* was selected for further functional validation.

**FIG 5:**
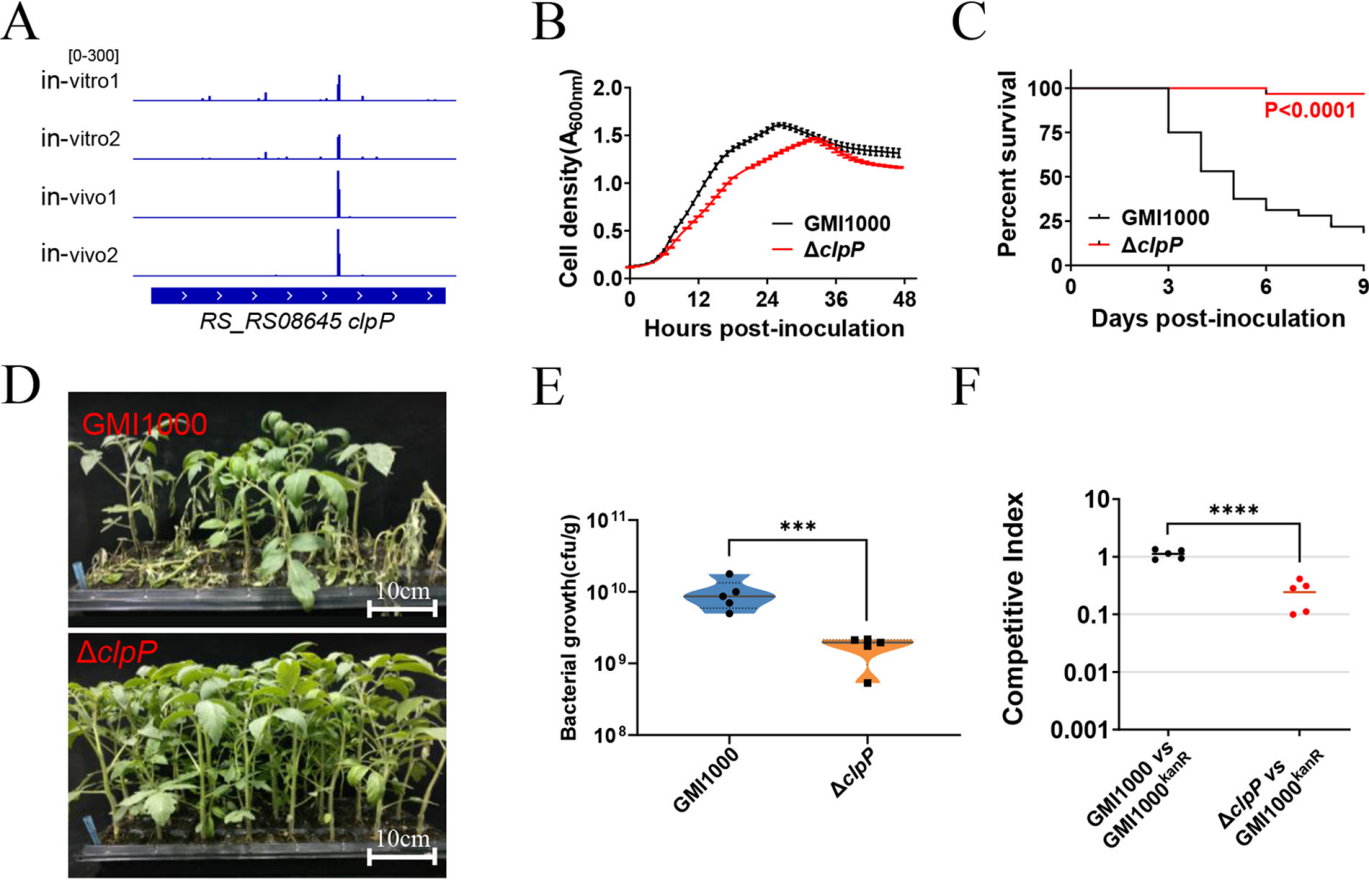
*clpP* is required for survival in tomato plants. (A) Transposon insertion distribution within *clpP* of transposon insertion libraries *in vivo* and *in vitro*. (B) Growth of the Δ*clpP* mutant and wild-type strain GMI1000 in BG medium. (C) Survival curve of tomato plants inoculated with the Δ*clpP* mutant and wild-type strain GMI1000. Kaplan-Meier survival analysis with the Gehan-Breslow-Wilcoxon method was used to compare the pathogenicity between the mutant and wild-type strains. *P* values of <0.05 were considered significant. (D) The bacterial wilt symptom of tomato plants 9 days after inoculation with the Δ*clpP* mutant and wild-type strain GMI1000. (E) Colonization by the Δ*clpP* mutant. The CFU of R. solanacearum strains in 1 g tomato plant stem were counted 5 days postinoculation. Asterisks indicate significant differences (***, *P* < 0.001; *t* test). (F) *In vivo* competitive index of the Δ*clpP* mutant. The Δ*clpP* mutant and GMI1000^Kanr^ were coinoculated into the stems of tomato plants. The competitive index was measured 5 days postinoculation. Asterisks indicate significant differences (****, *P* < 0.0001; *t* test).

As shown in Fig. 5A, three unique insertions in *clpP* were identified from the *in vivo* library, and 12 unique hits were identified in *clpP* for *in vitro* treatment. The number of weighted reads on average for *in vivo* treatment is 12, whereas the number for *in vitro* treatment is 48. As reported in our previous study (27), the targeted deletion of *clpP* substantially slowed the growth of R. solanacearum in rich BG medium (Fig. 5B). A Δ*clpP* mutant was inoculated into tomato plant stems to assess the pathogenic role of ClpP. As shown in Fig. 5C and D, 81% of the tomato plants infected with wild-type strain GMI1000 wilted 9 days postinoculation, whereas almost all tomato plants infected with the Δ*clpP* mutant survived. The result indicates that ClpP protease is essential for the fitness of R. solanacearum. Moreover, colonization assays and competitive index assay confirmed that ClpP protease is required for R. solanacearum survival in tomato plants (Fig. 5E and F).

## DISCUSSION

Besides the genes required for survival in tomato plants, Tn-seq indicated that the interruption of two genes improved relative fitness *in vivo* compared with *in vitro*. The read ratio (*in vivo*/*in vitro*) of XRE family transcriptional regulator *RS_RS05450* was 2.4, which means that the environmental stress within tomato plants improved the relative abundance of the *RS_RS05450* mutant by 2.4 times (Fig. S4A and Table S1). Previous experimental evolution and reverse genetic approaches revealed that mutation of *RS_RS05450*, namely, *efpR*, is associated with fitness gain on bean, tomato, and other host plants (14, 28). EfpR acts as a global catabolic repressor in R. solanacearum, and the absence of *epfR* would expand metabolic versatility and improve the relative fitness competing with wild-type R. solanacearum strains (28).

10.1128/mSystems.00838-21.6 FIG S4 Interruption of *efpR* (*RS_RS05450*) and *RS_RS05405* resulted in improved relative fitness *in vivo* compared with *in vitro*. (A) Transposon insertion distribution within *efpR* and *RS_RS05405* of transposon insertion libraries *in vivo* and *in vitro*. (B) Growth of the Δ*RS_RS05405* mutant in BG medium. (C) Competitive index of the Δ*RS_RS05405* mutant *in vitro* and *in vivo*. The Δ*RS_RS05405* mutant and GMI1000^Kanr^ containing a kanamycin resistance gene were coinoculated into the stems of tomato plants, and the *in vivo* competitive index was measured 5 days postinoculation. The Δ*RS_RS05405* mutant and GMI1000^Kanr^ were coinoculated into BG medium, and the *in vitro* competitive index was measured 12 h (log phase) and 36 h (stationary phase) postinoculation. Asterisks indicate significant differences (**, *P* < 0.01; ***, *P* < 0.001; *t* test). Download FIG S4, TIF file, 0.3 MB. Copyright © 2021 Su et al. 2021 Su et al. https://creativecommons.org/licenses/by/4.0/ This content is distributed under the terms of the Creative Commons Attribution 4.0 International license.

Besides *efpR*, the transposon insertion in *RS_RS05405*, which encodes nonheme iron oxygenase ferredoxin subunit, improved the relative fitness of R. solanacearum in tomato plants compared with that *in vitro*. Nonheme-iron-dependent oxygenases catalyze various reactions in the biodegradation of xenobiotics and the biosynthesis of bioactive natural products (29). The relative abundance of the *RS_RS05405* mutant *in vivo* was 13.2 times higher than *in vitro* (Fig. S4A and Table S1). *RS_RS05405* was deleted to validate this finding. As shown in Fig. S4B, *RS_RS05405* deletion slowed the growth of R. solanacearum, but the final bacterial concentration of the Δ*RS_RS05405* mutant was greater than that of wild-type GMI1000. The pathogenicity of the Δ*RS_RS05405* mutant was assayed. However, no statistical difference was found between the pathogenicity of the Δ*RS_RS05405* mutant and the wild-type strain. The *in vivo* and *in vitro* competitive indexes of the Δ*RS_RS05405* mutant were then assayed. Mutant Δ*RS_RS05405* and GMI1000^Kanr^ strains containing a kanamycin resistance gene were coinoculated into the stems of tomato plants and rich BG medium. R. solanacearum strains were recovered from tomato plants 5 days postinoculation. The competitive index of the Δ*RS_RS05405* mutant *in planta* was 0.48, whereas the competitive fitness of the Δ*RS_RS05405* mutant at the stationary phase in BG was 0.34 (Fig. S4C). The competition assay indicated that *RS_RS05405* is required for the survival of R. solanacearum *in vivo* and *in vitro*, but this gene may be more critical for *in vitro* survival than *in planta* survival.

Lipopolysaccharide (LPS) is a vital component of Gram-negative bacterial outer membrane and protects bacteria from harsh environmental conditions (30). The LPS of pathogenic bacteria has critical roles in biofilm formation, host attachment, and colonization (31). The pathogenic roles of different forms of LPS in R. solanacearum were systematically characterized, and all 13 investigated LPS-defective mutants were unable to cause any disease symptom in tomato plants (32). Consistently, eight of these 13 genes were identified in the present study as essential for R. solanacearum survival in tomato plants. These eight genes were involved in LPS core biogenesis (*RS_RS02830*, *RS_RS04545*, and *RS_RS04550*), LPS O-antigen biogenesis (*RS_RS03460*, *RS_RS03465*, and *RS_RS11050*), LPS O-antigen ligation (*RS_RS11060*), and mannose metabolism (*RS_RS15365*). Our targeted deletion also verified that the sorting and localization system of lipoprotein RS_RS01965, as well as the functional known membrane protein RS_RS04475, is required for *in vivo* survival. These results highlighted the importance of the cell membrane for the pathogenicity and survival of R. solanacearum.

Tomato xylem sap is a nutritionally limiting habitat, and R. solanacearum needs to regulate its metabolism and alter xylem sap biochemistry to adapt to this niche (33–35). Most proteinogenic amino acids are present at micromolar concentrations in xylem sap, and many of them are limiting for the growth of R. solanacearum (34). The biosynthesis of tryptophan is required for the pathogenicity of R. solanacearum on tomato and tobacco. Moreover, the biosyntheses of methionine and leucine are important for survival in tomato but not in tobacco, based on chemical mutagenesis (36). Tn-seq in the present study revealed that 19 genes involved in amino acid transport and metabolism are required for survival in tomato plants. These genes encode enzymes involved in the biosynthesis of chorismite, tryptophan, serine, cysteine, methionine, phenylalanine, tyrosine, and lysine. A Tn-seq study of R. solanacearum cultured in xylem sap extracted from tomato plant was recently reported in a preprint (37). Although the *in planta* environment is dynamically changing and is different from the environment in extracted xylem sap (38), 12 of the mentioned 19 genes were identified by the Tn-seq study of R. solanacearum cultured in tomato xylem sap (37).

Moreover, *RS_RS12160*, which encodes phosphoadenylyl-sulfate reductase, and *RS_RS06745*, which encodes sulfate ABC transporter permease subunit CysT and was here classified in “inorganic ion transport and metabolism (P)” by COG, were associated with cysteine biosynthesis. *RS_RS12160* and *RS_RS06745* were also previously identified (37). Overall, all the *in vivo* essential amino acid biosynthesis pathways revealed in the present study were hit by the Tn-seq of R. solanacearum cultured in xylem sap. Although many studies inferred that the biosynthesis of amino acids is important for the pathogenicity and *in planta* survival of R. solanacearum, targeted verification studies are limited (35). The present study deleted *RS_RS01965* and *RS_RS04475* in-frame to generate auxotrophic strains and validated that the biosyntheses of tryptophan and serine are essential for the fitness of R. solanacearum in host tomato plants.

Most of the known virulence factors of R. solanacearum are megaplasmid borne, including the type III secretion system, which delivers type III effectors into plant cells and is the key for pathogen and plant interaction (5), type VI protein secretion systems, flagellar motility determinants, chemotaxis genes, and EPS biosynthesis genes (10). However, most (117/132) of the *in vivo* survival genes identified in our study are chromosome borne, and most of the known virulence factors mentioned were not hit by the present study. Secreted virulence determinants, including type III effectors, type VI proteins, extracellular enzymes, and EPS, serve as public goods for the pathogenic population. Mutants can survive without the secretion of these virulence determinants by benefiting from the public goods secreted by other strains (39, 40). This may explain why these virulence genes were not identified by Tn-seq based on relative fitness. R. solanacearum is virtually nonmotile in plants, and motility and chemotaxis contribute to the early stages of host plant invasion and colonization (41). The transposon insertion library of R. solanacearum was directly injected into the stems of tomato plants in this study. Mutants without motility or chemotaxis could survive inside the stems of tomato plants. Future studies may inoculate the transposon insertion library of R. solanacearum via different methods to identify genes essential for the different stages of R. solanacearum infection.

## MATERIALS AND METHODS

### Bacterial growth conditions.

R. solanacearum strains were cultured in BG medium (10 g/liter Bacto peptone, 1 g/liter Casamino Acids, 1 g/liter yeast extract, and 5 g/liter glucose) or on BG agar medium at 28°C, except where noted otherwise. Escherichia coli strains were cultured in Luria-Bertani (LB) medium or on LB agar medium at 37°C. A final concentration of 25 μg/ml kanamycin was added to the medium when needed. The growth (*A*_600_) of R. solanacearum strains with three biological repeats was monitored every hour via Bioscreen C Pro (Oy Growth Curves Ab Ltd., Turku, Finland) to obtain growth curves. The Δ*RS_RS04490* mutant was cultured in Fahraeus medium (21, 42) with or without 17.5 mM serine to assay the auxotroph. The Δ*RS_RS09970* mutant was cultured in Fahraeus medium with or without 0.4 mM tryptophan to assay the auxotroph.

### Tn-seq of R. solanacearum in tomato plants.

The near-saturated transposon insertion library of R. solanacearum GMI1000 preserved at −80°C was adjusted to 1 × 10^8^ CFU/ml in BG medium and reactivated at 28°C for 1 h. The activated transposon insertion library was injected into the stems of 4-week-old tomato cultivar ‘Zhongshu No. 4’ plants cultured in a classic greenhouse. About 10 μl of transposon insertion library was inoculated per plant. The transposon insertion library before plant inoculation was used as the control group and referred to as “*in vitro*.” Transposon insertion libraries were recovered from plants 5 days postinoculation, when most plants showed a disease index of 3 or 2. The bacteria were recovered from 32 diseased plants and pooled for further steps to reduce the bottleneck of injected transposon insertion library and enrich bacterial DNA. The recovered transposon insertion library was referred to as “*in vivo*.” Total DNAs of the transposon insertion libraries before and after infection were extracted and divided into two groups for technical replicates. The total DNA samples were subjected to MmeI digestion, adapter ligation, and PCR amplification to construct Illumina sequencing libraries and subjected to sequencing and raw data preprocessing on the Galaxy web platform as previously described (20, 43). The index sequences of the sequencing manufacturer and transposon sequence in raw reads were trimmed. The reads were then separated based on the barcode for each sample, followed by barcode sequence trimming and read filtering by quality.

The preprocessed reads were mapped to the genome of R. solanacearum GMI1000 (GCA_000009125.1) by using Bowtie tolerating a 0-bp mismatch. The bam files from mapping were subjected to sample correlation coefficient computation and visualization via deepTools (44). We set the transposon insertion library before infection (*in vitro*) as the control, and gene essentiality *in vivo* was analyzed by TSAS, a Tn-seq analysis software (45). TSAS was run in two-sample analysis mode, while the other key input parameters were left in default. Ratio_reads (*in vivo*/*in vitro*) values of <0.5 with adjusted *P* (proportions_reads) values of <0.01 were set as the threshold values to identify genes required for survival in tomato plants (Table S1).

The transposon insertion distribution in targeted genes was visualized by Integrative Genomics Viewer using the wig files from TSAS as input (46). The COG categories of genes required for R. solanacearum survival in tomato plants were annotated by eggnog-mapper (47). The pathways of genes classified in amino acid transport and metabolism were analyzed by mapping in the KEGG pathway database and rendered by Pathview (48).

### Gene deletion in R. solanacearum.

The gene of interest was deleted in frame as previously described (27). Briefly, two flanking regions of the target gene were amplified using the primers listed in Table S2. These two DNA fragments were ligated via overlapping PCR and cloned into suicide plasmid pK18mobsacB or directly cloned into pK18mobsacB via three appropriate restriction enzyme sites. The recombined plasmid was verified by Sanger sequencing and imported into R. solanacearum wild-type strain GMI1000 by electroporation. The resultant recombinant strain was then cultured in a modified BG medium in which glucose was replaced by 10% sucrose for the second homologous recombination. The mutant that lacks the target gene was selected by PCR using the primers that flank the target gene (1F and 2R in Table S2).

10.1128/mSystems.00838-21.2 TABLE S2 Primers used in this study. Download Table S2, XLSX file, 0.01 MB. Copyright © 2021 Su et al. 2021 Su et al. https://creativecommons.org/licenses/by/4.0/ This content is distributed under the terms of the Creative Commons Attribution 4.0 International license.

### Pathogenicity phenotyping of R. solanacearum.

Susceptible tomato cultivar ‘Zhongshu No. 4’ was seeded and transplanted into 32-cell plug trays growing in a greenhouse. The 4-week-old plants were used for pathogenicity assay of R. solanacearum by stem injection following the protocol described elsewhere (21). The tested R. solanacearum mutant and wild-type strains were cultured in BG medium to the log growth phase and adjusted to 1 × 10^7^ CFU/ml with sterilized H_2_O. About 10 μl of adjusted bacterial suspension was injected into the stem 0.5 cm above the cotyledons using a disposable microsyringe. The wilting symptoms of inoculated plants were scored on a visual scale of 0 (no symptoms) to 4 (complete wilting) once per day. The 32 plants in a tray were inoculated for each R. solanacearum strain. Kaplan-Meier survival analysis with the Gehan-Breslow-Wilcoxon method was used to compare the pathogenicity between the mutant and wild-type strains (49). A *P* value of <0.05 was considered significant. The pathogenicity was assayed at least three times, and one representative result was presented.

The colonization of R. solanacearum strains was measured according to the previously described protocol (21). We sampled and weighed 1 g of the stem 1 cm above the inoculation point at 5 days postinoculation. The sampled stem was then sterilized in 70% ethanol for 30 s, rinsed in sterile water for 30 s, and ground in sterile mortars. The bacterial concentration was determined by plating on BG agar medium after serial 10× dilutions and expressed as CFU per gram of fresh stem. The bacterial concentrations of five infected tomato plants were measured for each R. solanacearum strain. The colonization differences between mutant and wild-type strains were analyzed with an unpaired *t* test.

Each R. solanacearum strain for the test was mixed with GMI1000^Kanr^ containing a kanamycin resistance gene at the same bacterial concentration and the same volume to assay the competitive index. The amount of R. solanacearum strains was determined by plating on BG medium with or without 25 μg/ml kanamycin. The amount of GMI1000^Kanr^ was determined by enumerating CFU on BG agar medium with added kanamycin, and the amount of the tested R. solanacearum strain was the number of CFU on BG agar medium without kanamycin minus the amount of GMI1000^Kanr^. The mixed bacterial suspension was then inoculated into tomato plants by stem injection. The amount of R. solanacearum strains in infected tomato plants was redetermined 5 days postinoculation. Competitive index was calculated as [tested strain CFU/GMI1000^Kanr^ CFU (5 days postinoculation)]/[tested strain CFU/GMI1000^Kanr^ CFU (before inoculation)]. Five biological replicates were performed for each R. solanacearum strain. The competitive index differences between mutant/GMI1000^Kanr^ and GMI1000/GMI1000^Kanr^ were analyzed with an unpaired *t* test.

### Data availability.

The processed reads and raw reads are available in the SRA database of NCBI (PRJNA766096). The wig files from TSAS were deposited in Figshare (https://doi.org/10.6084/m9.figshare.14220053).
