# Supplementary figures and images for: Genome-Wide Identification of Ralstonia solanacearum Genes Required for Survival in Tomato Plants
Source: mSystems. 2021 Oct 12;6(5):e00838-21. doi: 10.1128/mSystems.00838-21 (PMC8510521; doi:10.1128/mSystems.00838-21)

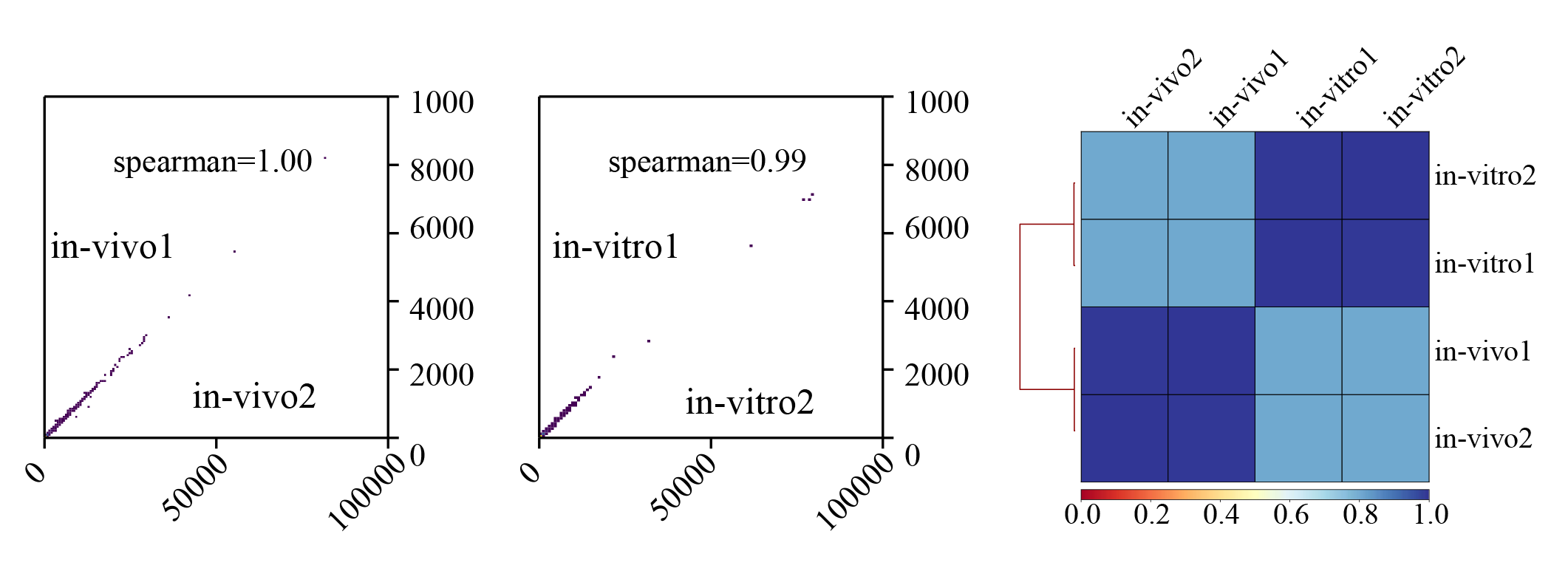

Supplement: FIG S1 [file msystems.00838-21-sf001.tif]

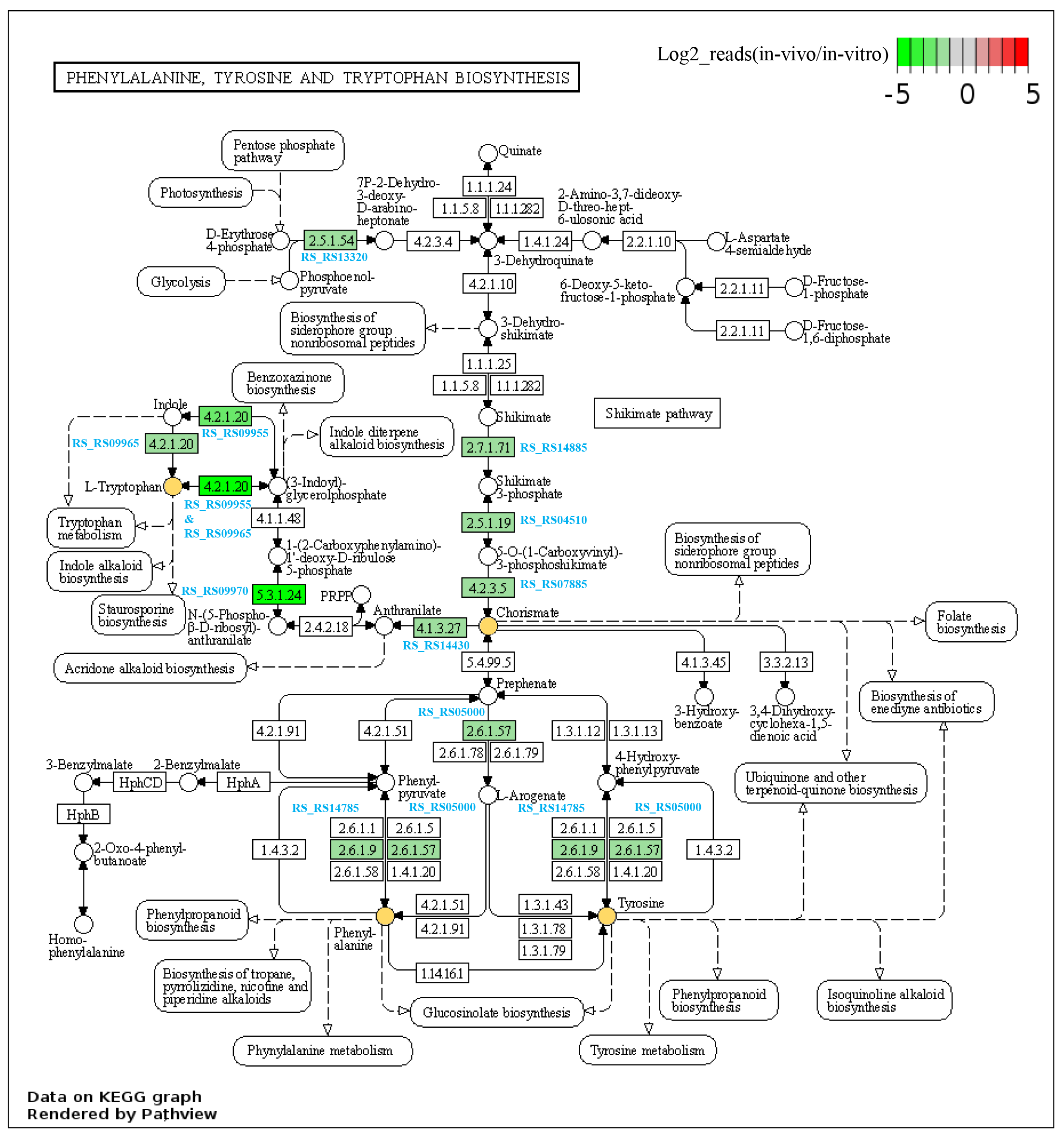

Supplement: FIG S2 [file msystems.00838-21-sf002.tif]

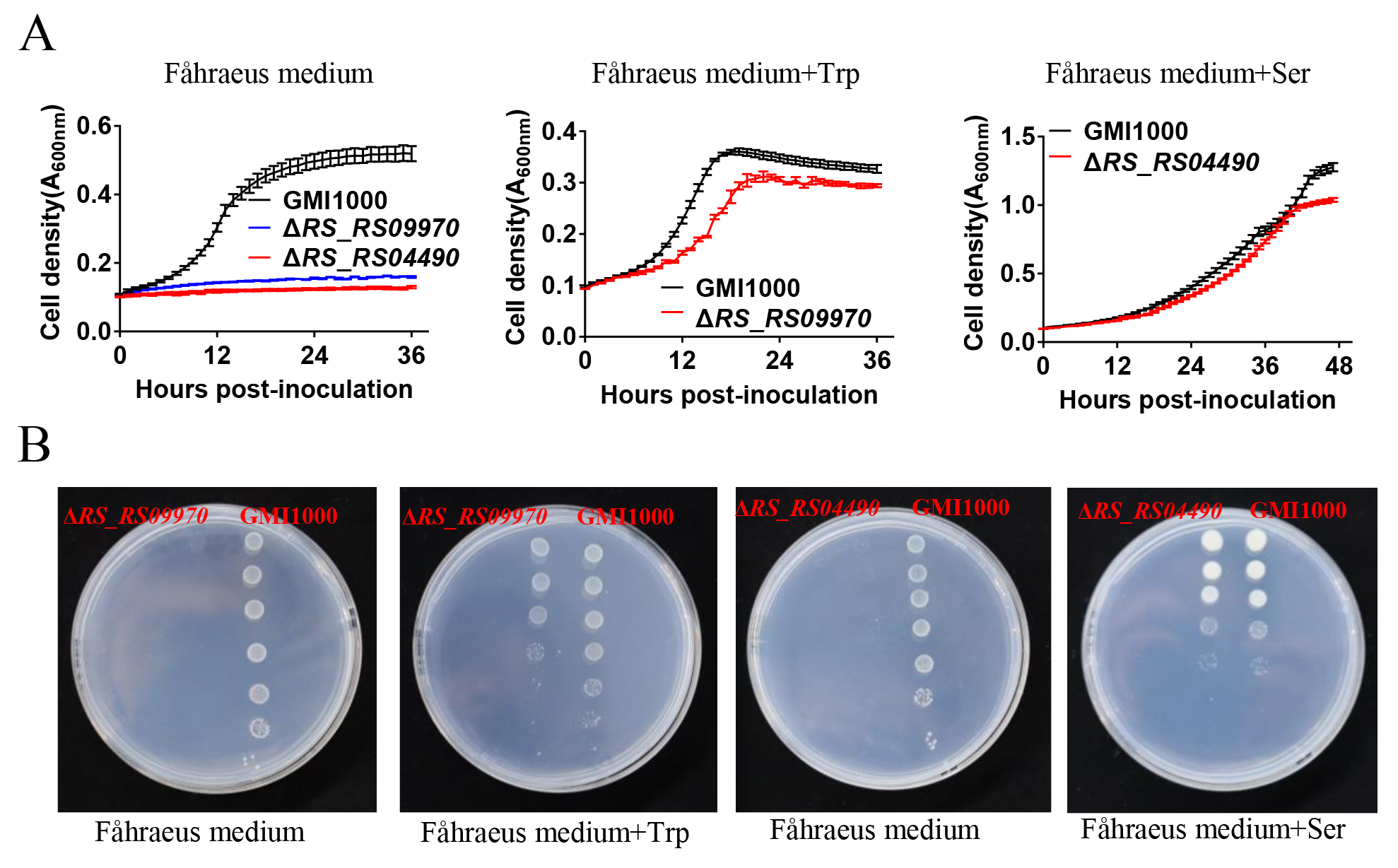

Supplement: FIG S3 [file msystems.00838-21-sf003.tif]

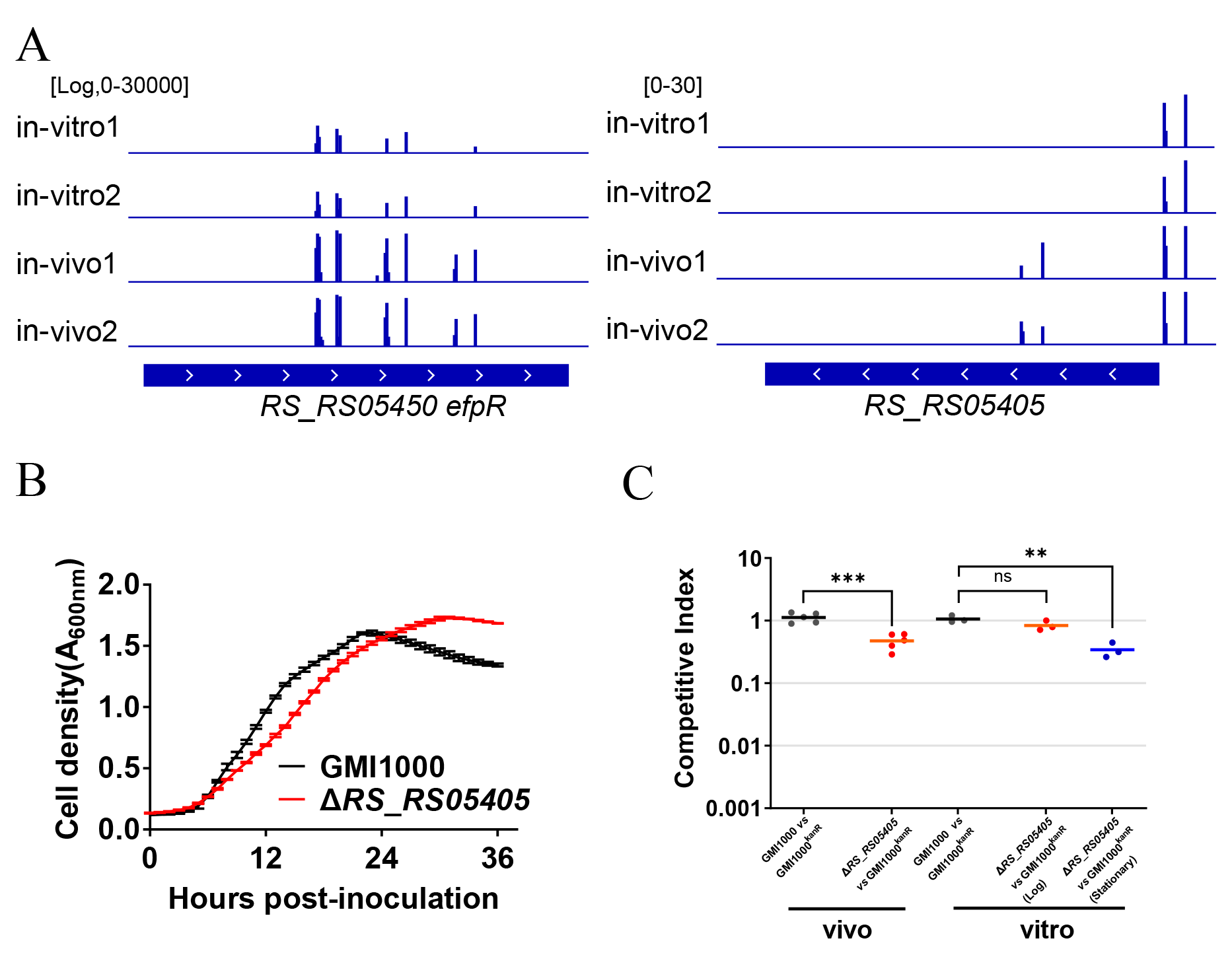

Supplement: FIG S4 [file msystems.00838-21-sf004.tif]
